# Perceptions of Community Health Workers (CHWs/PS) in the U.S.-Mexico Border HEART CVD Study

**DOI:** 10.3390/ijerph110201873

**Published:** 2014-02-10

**Authors:** Hector G. Balcazar, Sherrie Wise, Alisha Redelfs, E. Lee Rosenthal, Hendrik D. de Heer, Ximena Burgos, Maria Duarte-Gardea

**Affiliations:** 1 School of Public Health, University of Texas Health Science Center, Houston, El Paso Regional Campus, 1101 N. Campbell CH 410, El Paso, TX 79902, USA; E-Mails: sherrie.b.wise@uth.tmc.edu (S.W.); alisha.hayden@uth.tmc.edu (A.R.); 2 Project on Community Health Worker Policy and Practice, Institute for Health Policy, University of Texas School of Public Health, c/o El Paso Regional Campus, 1101 N. Campbell, CHS 410, El Paso, TX 79902, USA; E-Mail: Lee.Rosenthal@uth.tmc.edu; 3 Department of Physical Therapy and Athletic Training, Northern Arizona University, P.O. Box 15105, 208 E Pine Knoll Dr., Flagstaff, AZ 86004, USA; E-Mail: dirk.deheer@nau.edu; 4 Department of Public Health Sciences, The University of Texas at El Paso, 500 W. University Ave., El Paso, TX 79968, USA; E-Mails: xburgos@miners.utep.edu (X.B.); moduarte@utep.edu (M.D.-G.)

**Keywords:** community health worker, Hispanics, cardiovascular disease, community health resources

## Abstract

Although prior research has shown that Community Health Workers/Promotores de Salud (CHW/PS) can facilitate access to care, little is known about how CHW/PS are perceived in their community. The current study reports the findings of a randomized telephone survey conducted in a high-risk urban community environment along the U.S.-Mexico border. In preparation for a community-based CHW/PS intervention called the HEART ecological study, the survey aimed to assess perceptions of CHW/PS, availability and utilization of community resources (recreational and nutrition related) and health behaviors and intentions. A total of 7,155 calls were placed to complete 444 surveys in three zip codes in El Paso, Texas. Results showed that participants felt that healthful community resources were available, but utilization was low and variable: 35% reported going to a park, 20% reported having taken a health class, few reported using a gym (12%), recreation center (8%), or YMCA/YWCA (0.9%). Awareness and utilization of CHW/PS services were low: 20% of respondents had heard of CHW/PS, with 8% reporting previous exposure to CHW/PS services. Upon review of a definition of CHW/PS, respondents expressed positive views of CHW/PS and their value in the healthcare system. Respondents who had previous contact with a CHW/PS reported a significantly more positive perception of the usefulness of CHW/PS (*p* = 0.006), were more likely to see CHW/PS as an important link between providers and patients (*p* = 0.008), and were more likely to ask a CHW/PS for help (*p* = 0.009). Participants who utilized CHW/PS services also had significantly healthier intentions to reduce fast food intake. Future research is needed to evaluate if CHW/PS can facilitate utilization of available community resources such as recreational facilities among Hispanic border residents at risk for CVD.

## 1. Introduction

Community health workers (CHWs) or promotores de salud (PS) are community members who serve as frontline public health workers, facilitating access to health and social services for those in need. CHW/PS have been shown to be able to perform important roles at many ecological levels, including advocacy at the local, state, and national levels [1,2,3,4,5,6,7,8,9], social support, health education, service coordination and referral at the individual level, interpersonal, and community levels [1,2,3,4,6,7,8,9]. The changing nomenclature in regards to CHW/PS now includes “experience-based experts” [8,9], and in 2010 CHW/PS were recognized in the Standard Occupational Classification [10]. Recently, CHW/PS services have been utilized to deliver a range of evidence-based cardiovascular risk reduction interventions in Hispanic communities along the U.S.-Mexico border [1,2,3,4,11,12,13]. To date, however, few programs have examined how community members perceive the CHW/PS approach, and whether exposure to CHW/PS services is associated with utilization of healthful resources in the community environment. Future initiatives aimed at addressing risk factors of common public health problems through CHW/PS approaches would benefit from knowledge about how CHW/PS are received.

The 2009–2013 Health, Education, Awareness, Research, Team (HEART) ecological study was a community-based participatory research project for Hispanic communities aimed at CVD prevention. The project was implemented at the U.S.-Mexico border and was facilitated by CHW/PS [1,2,3,4]. Within this ecological approach, HEART explored how various levels of the environment, and individuals within that environment, support a reduction of detrimental lifestyle behaviors affecting CVD among Hispanics. The HEART project was guided by a conceptual framework developed for many of the intervention programs implemented in El Paso, Texas utilizing CHW/PS [1,2,3,4,11,13,14,15]. This conceptual framework described how psychosocial pathways of behavior change can lead to healthier lifestyles and CVD risk reduction among Mexican Americans. 

Within the HEART ecological study a phone survey was conducted as one baseline community assessment strategy to evaluate utilization and perceptions of CHW/PS services, behavior and intentions. This survey provided the background for an intervention aimed at matching available community resources (e.g., parks, community centers, YMCA facilities) with opportunities for high risk community members to utilize these resources, and ultimately promoting pathways of behavioral change as proposed by our conceptual framework [14,15,16]. The objective of this article is to report the findings of the randomized phone survey and answer the following primary questions: (1) What is the level of public awareness and utilization of CHW/PS?; (2) what is the perception of CHW/PS usefulness and importance in community-based settings such as recreational facilities; and are these perceptions different across level of utilization? (3) what is the perception of the availability of healthful community resources including exercise infrastructure and healthy foods for purchase? and (4) is there a relationship between previous utilization of CHW/PS services and health behaviors (diet, exercise, substance use and health screenings) and intentions? 

## 2. Methods

### 2.1. Study Setting

A community telephone survey was conducted in three zip codes of the urban lower valley area of El Paso, Texas as a baseline community-level evaluation tool of HEART [4]. Two of the zip codes served as the site of the HEART intervention after this survey was conducted. The purpose of the survey was to determine baseline health behavior as well as perception and utilization of CHW/PS and other resources for healthy living. Selected zip codes had comparatively low socioeconomic indicators. Median household income of the three areas were $26,447, $23,400, and $26,650, approximately one-half the national median of $41,994 [17]. The percentage of individuals below the poverty line in all three zip codes was 27.2%, 28.6%, and 30.9%, approximately twice the national percentage (12.4%). The percentage of those aged 25 years and over having completed high school or higher was also comparatively low at 52.7%, 50.4%, and 51.0% in relation to the national percentage of 80.4%. Furthermore, of the same zip codes, those having completed a bachelor’s degree represented one-fifth the national percentage (24.4%), at 5.3%, 5.9%, and 5.8% [17]. The percentage of Hispanics represented in these zip codes is on average around 94% as compared with 81.4% for the city of El Paso [17]. Sociodemographic data of the zip codes utilized in this study very closely mirrors the data from the entire City of El Paso. 

### 2.2. Study Design

Cross-sectional baseline data from the phone survey was collected between 6 October and 12 November of 2009 from all three zip codes. The Institute for Policy and Economic Development located at The University of Texas at El Paso was hired to conduct the survey. The target number was to achieve an average of 150 participants per zip code to reach sufficient variability of responses. Between the hours of 3:00 p.m. and 7:30 p.m. during weekdays and weekends, trained, bilingual surveyors contacted respondents from 8,000 randomly selected phone numbers across the three zip codes. The times and days were selected as a way to maximize opportunities to include both men and women, employed and unemployed respondents. The first week served as a training week consisting of practicing and pilot testing the survey script, which consisted of an introduction, an explanation why participants were selected (zip code), and the survey topic (described as questions about health behavior and awareness). It was emphasized that participation was voluntary and responses confidential. A total of 7,155 attempts were required to achieve 444 completed surveys. Approval was obtained from both institutional review boards of The University of Texas at El Paso and the University of Texas Health Science Center Houston, School of Public Health.

### 2.3. Measures

The bilingual (English/Spanish) survey consisted of 56 questions, was approximately 23 min in duration, and measured demographics including language spoken at home and health insurance, health behavior and intentions, as well as awareness of and utilization of existing community resources for healthful living. The survey was based on questions adapted from existing surveys including the CDC Behavioral Risk Factor Surveillance System Questionnaire [18], the CHW section of the American Public Health Association (APHA), and previously validated surveys in this population [4]. 

#### 2.3.1. Availability and Utilization of Healthful Community Resources

Perception of community resources including parks and gyms, community programs that teach about healthy living, and places in the community where healthy foods available for purchase were measured using a 10-point Likert scale (1 Strongly disagree; 10 Strongly agree). The utilization measure included a yes/no response for the following two constructs of previous utilization of CHWs as measured by household contacted by a CHW/PS and ever referred to a CHW/PS by a healthcare provider.

#### 2.3.2. Health Behaviors and Intentions

Behavior and intention questions were based on the HEART participant questionnaire [4] and the BRFSS [18]. Dietary behaviors were measured using a 10-point Likert scale (1 Strongly disagree to 10 Strongly agree) for the following: (1) buy and prepare healthy foods for my family, (2) cook using foods lower in fat, (3) cook using foods lower in salt, (4) eat at least five servings of fruit and vegetables per day, and (5) limit the consumption of fast food to no more than once per week. 

Exercising a minimum of thirty minutes three times a week was measured using yes/no responses, and utilization of community resources for exercise was measured by asking: “In the past 6 months have you gone to any of the following places for the specific purpose of exercising?” Respondents were asked to select one or more places from a list of nearby locations. Smoking behavior and binge drinking in the past month was also measured. 

Intent to engage in the following behaviors in the next six months was measured using a 10-point Likert scale: (1) eat at least five servings of fruit and vegetables per day, (2) limit the consumption of fast food to no more than once per week, (3) reduce alcohol intake, (4) participate in a class that teaches healthy living and (5) exercise 30 min at least three times a week.

#### 2.3.3. Awareness, Perceptions, and Utilization of Community Health Workers

Familiarity with and perception of CHWs was measured through seven questions; before being asked questions, respondents were given the following definition of CHW/PS excerpted from the CHW Section of the American Public Health Association:

“Community health workers are community members who serve as frontline public health workers, and who serve as liaisons between health and social services and the community to facilitate access to services and service delivery, including health education and promotion services.”

Awareness of CHW/PS was measured using a yes/no response as was utilization; utilization questions were: “*Has a doctor or other healthcare provider referred you to a Community Health Worker/Promotora de Salud (CHW/PS) before*?” and “*Has someone in your household been contacted by a Community Health Worker/Promotora de Salud (CHW/PS) before*?” 

Respondents were then asked to provide their impression of CHW/PS usefulness using a 10-point Likert scale (1 Not useful at all; 10 Very useful) in response to the following two questions: “*What is your impression of how useful Community Health Workers could be*?” and “*If I had a question regarding my health or the health of a family member, I would ask a Community Health Worker/Promotora de Salud for help*.” 

Finally, two questions were used to determine perceived importance (on a 10-point Likert scale) of CHW/PS in clinical settings as well as community based organizations. They were: “*I think that, in a clinical setting (such as hospitals, and health centers), Community Health Workers/Promotoras de Salud are an important link between the healthcare providers and the patients*,” and “*I think that, in a community-based organization (such as gymnasiums, parks and recreation facilities), Community Health Workers/Promotoras de Salud are an important link between the organization and the community they are trying to reach.*”

### 2.4. Statistical Analyses

Descriptive statistics and frequencies were used to characterize the information on socio-demographic indicators, utilization of community resources, perceptions and utilization of CHW/PS services, and participant health behaviors and intentions. Due to several slightly skewed and/or kurtotic variables, non-parametric methods were used. To compare perceptions, behaviors and intentions between those participants who utilized CHW services to those who did not, Kruskall-Wallis one-way analyses of variance were conducted. A Bonferroni correction was applied to correct for the number of comparisons. All analyses were conducted with SPSS version 19.0 (SPSS Inc., Chicago, IL, USA).

## 3. Results

### 3.1. Socio-Demographic Characteristics

Socio-demographic characteristics are presented in Table 1. Data are presented for the total sample without stratifying by zip code. Close to 50% of respondents reported they speak Spanish at home, with the remainder speaking English or both, confirming the bicultural-bilingual nature of this border sample of residents. Half of the respondents completed the survey in Spanish. Close to 40% indicated they did not have health insurance coverage. The majority of respondents were women (77%) and 55% of the sample had and age of 50 years or older and the median years living in El Paso was 30.

**Table 1:** Demographic Characteristics of Survey Participants.

*N* = 444 Respondents		*N*	%
Age			
	18 to 29	64	15
	30 to 49	131	30
	50+	243	55
Sex			
	Male	103	23
	Female	341	77
Household Language			
	Spanish	214	48
	English	88	20
	Both	140	32
Language of Survey Conduction			
	Spanish	230	52
	English	214	48
Health Insurance			
	None	159	36
	Private	107	24
	Medicare	104	24
	Medicaid	33	7
	Other	38	9
Doctor’s Visit Within Past Year			
	Yes	324	73
		*Median*	*SD*
Years in El Paso County		30	19

### 3.2. Public Awareness, Utilization and Perceptions of Usefulness of CHW/PS

To address research questions 1 and 2, results of perceptions and utilization of community resources for healthful living, including CHW/PS are presented in Table 2. Only 20% of respondents had heard of CHW/PS. Of all respondents, a total of 8% reported CHW utilization (either having been referred to CHW/PS by a doctor or healthcare provider, and/or ever having been contacted by CHW/PS). In spite of this low percentage of CHW/PS utilization, all respondents taken together had a positive impression of CHW usefulness (median 9.0), and in CHW importance in linking healthcare providers and patients (median 8.0) and community-based organizations and the community (median 8.0). Respondents also expressed high intentions (median 8.0) to ask CHW/PS for their help if a question arises regarding their own health or a family members’ health.

Kruskall Wallis (KW in Table 2) one-way analysis of variance was used to compare participants who had utilized CHW/PS services to those who had not on each of the variables. These analyses showed that respondents who had utilized CHW/PS services evaluated their usefulness as significantly higher (*p* = 0.006). They were further significantly more likely to ask a CHW/PS for help (*p* = 0.009) and were more likely to see CHW/PS as an important link between providers and patients in a clinical setting (*p* = 0.008). 

**Table 2:** Perceptions and Utilization of Community Resources for Healthful Living.

Perceptions and Utilization Variables	Median ^1^	SD	KW ^2^
**Perceptions (and awareness) of CHW services** Impression of usefulness of CHW/PS (*N* = 430) ^3^	9.0	2.5	0.006 *
Would ask a CHW/PS for help for questions about own/family member’s health (*N* = 434)	8.0	3.1	0.009 *
CHW/PS are an important link between providers and patients in clinical settings (*N* = 431)	8.0	2.8	0.008 *
CHW/PS are an important link between community–based organizations and the community (*N* = 426)	8.0	2.8	0.171
**Perceptions of Community Resources for Healthful Living**			
Feel there are places in community that teach healthy living (*N* = 427)	8.0	3.0	**0.027**
Feel there are places in my community where healthy foods are available (*N* = 428)	9.0	2.3	0.617
Feel there are places in community that I can go exercise (*N* = 426)	9.0	2.9	0.560
Buy and prepare healthy foods for my family (*N* = 274)	9.0	1.8	0.211
**Utilization of Community Resources for Healthful Living**	***n***	**%**	**KW**
Ever heard of CHW/PS	87	20	N/A
Household contacted by a CHW/PS (*N* = 436)	22	5	--
Ever referred to a CHW/PS by health provider (*N* = 435)	19	4	--
Taken a class to improve my health past 3–6 months	86	20	0.603
Gone to any of the following places to exercise in past 6 months:			
Have not gone to any place	108	24.3	N/A
Gym	57	12.8	N/A
Recreation center	38	8.6	N/A
City park	159	35.8	N/A
YWCA/YMCA	4	0.9	N/A
Any other place	154	34.7	N/A

^1^ based on likert scale: 1 = Strongly disagree; 10 = Strongly agree; ^2^ Krusall-Wallis (KW) one-way analysis of variance comparing participants who had previously utilized CHW/PS versus those who did not. Bolded values indicate statistical significant Kruskall-Wallis. * Statistically significant after applying Bonferroni correction (shown with an asterisk). ^3 ^ For those not familiar with CHW/PS, impression was based on definition of CHW/PS read to survey respondents. *N* = 444 unless otherwise noted.

### 3.3. Perception of the Availability of Healthful Community Resources

Respondents believed that the environment where they live has programs available that teach about healthy living (median 8.0), that there are places available in the community to purchase healthy foods (median 9.0), and that there are places that they can go to exercise (median 9.0). They also felt confident that they could buy and prepare healthy foods for the family (median 9.0). 

A total of 20% reported having taken a class to improve their health in the last 3–6 months. Small proportions reported having gone to a gym (12%), recreation center (8%) or YMCA/YWCA (0.9%) to exercise. The most frequently visited locations for physical activity were city parks (35% of respondents). Taking into account a Bonferroni correction, there were no significant differences between participants who had utilized CHW/PS services compared to those who did not.

### 3.4. Health Behaviors and Intentions

Table 3 describes the self-reported percentages of respondents’ health behaviors for exercise, smoking, binge drinking, and for screenings for diabetes, cholesterol and blood pressure. Respondents had a median score of 6.0 (*n* = 430) (on the 10-point scale) agreeing that they ate at least 5 servings of fruits and vegetables per day, a median score of 9.0 in terms of limiting how often they eat fast food to no more than once per week, and a mean score of 8.0 of cooking using food low in salt and fat. 

**Table 3:** Health Behavior and Intentions.

Dietary Behavior Variables	Median ^1^	SD	KW ^2^
Cook using foods lower in fat (*n* = 274)	8.0	2.1	0.901
Cook using foods lower in salt (*n* = 274)	8.0	2.3	0.705
Eat at least 5 fruits and vegetables a day	6.0	2.9	0.101
Limit fast food to no more than once a week	9.0	3.1	0.364
**Lifestyle and Screening Behavior Variables **	***n***	**%**	**KW**
Exercise 30 min 3 times a week	253	79	0.746
Exercise in the past month	250	66	0.131
Smoking	39	9	0.616
Binge drinking past month	66	15	0.679
Cholesterol screening past 2 years	323	75	0.159
Blood pressure screening past 2 years	355	83	0.838
Diabetes screening past 2 years	306	71	0.175
**Behavioral Intention Variables **	**Median**	**SD**	**KW**
Intends to eat 5 servings of F/V a day within 6 months	8.0	2.3	0.648
Intends to limit fast food to no more than once a week within 6 months	10.0	2.6	0.009 *
Intends to reduce alcohol intake within 6 months	10.0	3.7	0.024
Intends to participate in healthy living class within 6 months	8.0	3.2	0.419
Intends to exercise 30 min 3 times a week within 6 months	10.0	2.4	0.175

^1^ Based on Likert scale: 1 = Strongly disagree; 10 = Strongly agree; Bolded statistically significant; * Statistically significant after applying Bonferroni correction. ^2^ Kruskall-Wallis (KW) one-way analysis of variance compared participants who had previously utilized CHW/PS versus those who did not. *N* = 444 unless otherwise noted.

For all respondents (*n* = 444), the median intention to take part in a class to improve their health within six months was 8.0 (±3.2). More than 70% of the sample reported having had a screening for cholesterol, blood pressure and diabetes in the past 2 years. The percentage of smoking was 9%, and binge drinking in past month was reported by 15% of the participants. Finally, more than 60% of the sample reported having performed some form of exercise per week or in the past month.

Respondents with previous utilization of a CHW/PS showed significantly greater intentions to perform a small number of health behaviors in the next six months than respondents without prior history of utilization. These included (1) intention to limit fast food intake to no more than once per week (*p* = 0.009), and (2) intention to reduce alcohol intake (*p* = 0.024), although this behavior is not significant after applying a Bonferroni correction. 

## 4. Discussion

This study aimed to assess the awareness and perception of the utility of community health workers (CHW/PS) as a health and support resource component for the community. We found that community respondents have little knowledge of and contact with CHWs, but that perception of CHW usefulness was high. Respondents who had previously utilized CHW/PS (were either referred to a CHW/PS or their household was contacted by CHW/PS) showed significantly more positive perceptions regarding the usefulness of CHW/PS as well as the importance of CHW/PS in clinical settings. Furthermore, these community respondents also felt more strongly that they would ask a CHW/PS for help if they had a question regarding their health or the health of a family member. Respondents with a history of CHW/PS utilization also intended to limit their fast food intake and reduce their alcohol use within the next six months.

Interestingly, this study reported a positive perception of the availability of a variety of community resources for healthy living. There are places to go to exercise, yet the facilities were not commonly used. The obvious question of possible barriers to utilization emerges, and may perhaps explain this disparity. While the environmental need for availability appears to be met, ancillary barriers are arguably the strongest predictor of health-promoting behavior (e.g., exercising regularly), and as such must be addressed for lifestyle interventions to be effective. A study of 328 Hispanic adults that addressed barriers to healthy living found that participants in a CHW/PS intervention improved health habits at follow-up [16]. 

This study found that utilization of available exercise resources such as gyms, recreation centers, and the YWCA was low. Utilization of city parks was the highest of all community resources. To address this, one of the objectives of the HEART Project is to increase utilization by placing CHW/PS in the YWCA, city parks, and city recreation centers [4]. Here, CHW/PS work to motivate respondents to prevent chronic disease, and provide related educational curriculum and support that has proven effective in previous interventions within Hispanic communities [4,11,13,19]. Furthermore, CHW/PS in this community are more commonly used in clinical settings than in community-based organizations (CBOs ) which could account for respondents’ higher perceived importance of CHW/PS in clinical settings than CBOs. One aim of HEART is to sustainably integrate CHW/PS into CBOs like YWCA as well as Parks and Recreation.

### 4.1. Limitations

This study had several limitations. Due to the cross-sectional nature of the survey, only associations are presented and temporality of any effects cannot be assessed. Selection bias due to self-selection of participation is part of this concern and may be due in part because the survey did not include individuals whose primary phone was a mobile phone. However, the implementation of the survey was systematic, following a strict protocol expected to limit biases from implementation or from the influence of the researchers on participant answers. Another limitation is the fact that respondents may have responded differently based on individual comprehension of the CHW/PS definition provided which presents a possible threat to statistical conclusion validity. However, based on the cumulative experience of our team working with Hispanic communities and the CHW/PS workforce, the finding that only 20% of respondents reported they had heard of CHW/PS and less than 4% had been referred to CHWs, we feel the social desirability bias is less of a concern with the utilization questions. *P*-values reported probably understate the true risk of type 1 error due to exploratory nature of tests. As this survey was recently developed, the degree to which we may generalize the results to other groups or settings is as yet unestablished. In the future, cross validation with other samples will provide more confidence in the external validity of the measure and these findings. 

### 4.2. Implications for Practice

As this baseline study showed, although respondents seem to feel that healthful resources are available, there is substantial room for improvement in terms of taking advantage of these resources. CHW/PS can serve multiple functions when linking the public to healthful community resources [20]. For example, they can serve as agents for community outreach to increase public awareness of community resources and facilitate the utilization of these resources when possible [6]. If community resources are available for healthy living, CHW/PS provide a great opportunity to facilitate utilization of these resources [21] and facilitate behavior change. 

Furthermore, CHW/PS can provide health education within the places available in the community for healthy living. In this way, CHW/PS don’t simply improve public utilization of resources for healthy living, they are themselves such resources [20,22]. The HEART project has previously shown how this process can be implemented [1,2,3,4,11]. However, navigation for the utilization of clinical services for screenings, and for control of health conditions can also be accomplished by CHW/PS [3,20] when they are integrated as part of a multidisciplinary workforce in clinic settings. The Patient Protection and Affordable Care Act is a potential key resource available for clinics and community-based organizations to include CHW/PS as part of their workforce to serve as a driving force in chronic disease prevention and control among individuals like those participating in this study [21].

## 5. Conclusions

CHW/PS are considered a valuable workforce that enhances opportunities for Hispanics at risk of developing CVD to take advantage of community resources already available to them. Establishing public awareness of available resources for healthy living is fundamental in increasing utilization of such resources. However, availability and public awareness is not enough. Through community outreach and health services navigation, CHW/PS can serve as links between the public and available resources. CHW/PS can also serve in both clinical and or community-based settings to provide health information, stimulate utilization of local facilities for exercise and healthy living, and improve lifestyle and screening behaviors among many roles [6,22].

Increased awareness of the role that CHW/PS can play in communities is warranted and must be part of new health promotion outreach models for reducing CVD risk among Hispanic populations living in the U.S. An ecological public health model of prevention that uses CHW/PS to link the public to existing community resources and programs for healthy living is a promising area of research and practice.
